# Editorial: Towards a Refined Understanding of Social Trust (T-R-U-S-T)

**DOI:** 10.3389/fnhum.2020.00305

**Published:** 2020-08-19

**Authors:** Frank Krueger, Andreas Meyer-Lindenberg

**Affiliations:** ^1^School of Systems Biology, George Mason University, Fairfax, VA, United States; ^2^Medical Faculty Mannheim, Central Institute of Mental Health, University of Heidelberg, Mannheim, Germany

**Keywords:** oxytocin, AVPR1A gene, gender, age, trust game

Social trust is an essential ingredient for nearly every aspect of our daily lives. A plethora of investigations have started to gain a deeper understanding of trust; however, a coherent conceptual framework that integrates separate findings into a *psychoneurobiological model of trust* is still lacking. As a joined effort, psychologists, economists, and neuroscientists submitted for this Research Topic *empirical* and *theoretical work* in the form of *original research, review*, and *opinion papers* to shed light on the *behavioral, psychological*, and *neural levels* of trust:

At the *behavioral level*, the research community commonly relies on the trust game (TG) paradigm as an incentivized measure of individual variability for both trust and trustworthiness behavior. Alos-Ferrer and Farolfi reviewed not only the strength and limitations inherent in this popular paradigm but also explored the relations to alternative instruments for future investigations.

At the *neuropsychological level*, experimental paradigms allow evaluating the impact of contextual, idiosyncratic, and demographic factors on the psychological components of trust (motivation, affect, and cognition) and the underlying neural mechanisms—for example, through functional magnetic resonance imaging. Fareri highlighted in his review the neurobehavioral mechanisms of trust and reciprocity through the lens of implicit and explicit social appraisal and learning processes—stressing to focus more on its underlying *neurocomputational mechanisms* in future studies.

Fairley et al. examined people's own, naturally occurring beliefs to explore the subsequent outcome of their choices—implementing a TG for social and a lottery for non-social contexts. Only trust decisions as investment amount in TG parametrically modulated anticipatory reward and outcome evaluation in the ventral striatum—demonstrating a novel approach for using people's inherent sets of beliefs for studying reward processing.

Although economic decision-making is commonly characterized as a rational phenomenon, real-world decisions are clearly influenced by affect. Eimontaite et al. investigated cooperation as a precursor of trust while participants played a Prisoner's Dilemma game under partner-directed sympathy, anger, and neutral emotion conditions. Left amygdala activity was indicative of emotion enhancement and increment of cooperative behavior, whereas the left putamen suppressed emotion to overcome anger and engage in cooperation under the influence of partner directed emotion.

People may change their behavior, sometimes against their personal preferences, according to the opinions of their peers. Wei et al. studied the effect of social influence on trust behavior. Participants conformed to others' opinions and behaviors in the TG—activating ventromedial prefrontal cortex (PFC) and ventral striatum—indicating that they felt rewarded confirming to other's opinions.

Parental investment and social role theories predict that men trust more to maximize resources, whereas women trust less due to a higher sensitivity to social risk. Wu et al. examined gender differences in trust by simultaneously scanning male and female same-gender, fixed dyads, who played a multi-round TG with varying levels of payoff as an indicator of risk. Men trusted more than women, and the payoff level moderated the effect of gender on trust behavior. Men demonstrated equivalent activation in the subgenual anterior cingulate cortex across the payoff level, whereas women showed a decreased activation with increasing payoff level—explaining women's higher risk to social risk.

Gender differences in trust and trustworthiness during adolescence is a key period of change in social behavior. Lemmers-Jansen, Fett, Shergill et al. studied age-related gender differences in trust and trustworthiness in adolescence, implementing multi-round TGs simulating a pre-programmed cooperative and an unfair partner. For repeated cooperative interactions, no gender differences were found but younger compared to older adolescents showed a slightly steeper increase of investments, whereas younger males reacted with a stronger decrease of investments than older males for unfair interactions. Those gender-by-age interactions on trusting revealed activity in temporoparietal junction and caudate—showing a stronger influence of age in males than in females during cooperative and the reverse in unfair interactions.

At the *neurochemical level*, exogenous administration of neuropeptide hormones (e.g., oxytocin, OXT) helps to reveal the neural signaling pathway mechanisms underlying trust behavior. Original landmark studies claiming a crucial role of OXT in enhancing trust have been questioned by subsequent meta-analytic approaches, large scale non-replications, or failure to reproduce findings in different contexts. Xu et al. argue in their review that OXT may play a key role in conforming to and learning from trusted individuals who are either in-group members and/or perceived experts instead of facilitating trust *per se*. Therefore, future studies should establish how motivational, affective, and cognitive aspects of trust interact with the effects of OXT on social learning and conformity.

At the *neurogenetic level*, the impact of single-nucleotide polymorphisms such as the OXT receptor (OXTR) gene on trust behavior have been studied. Nishina et al. examined whether the association between a common repeat length polymorphism in an intron of the arginine-vasopressin receptor 1A (AVPR1a) gene is associated with TG and attitudinal trust measures. Compared to their previous OXTR gene findings, this polymorphism of AVPR1a also revealed sex differences: men with a short form of AVPR1a not only trusted but also reciprocated more in the TG, but no associations with attitudinal trust were found. As a result, future studies should examine the underlying brain functions and structures mediating the association between AVPR1a and trust behavior.

Identifying the psychoneurobiological patterns of trust in healthy people can potentially shed light on trust impairment, as present in some *psychiatric disorders*. A prime candidate helping to build trust as the glue to positive social interactions could be social mindfulness—the ability and willingness to see and consider another person's needs and wishes during social decision-making. Lemmers-Jansen, Fett, Van Doesum et al. investigated whether first-episode psychosis patients (FEP) and patients at clinical high-risk (CHR) show reduced social mindfulness applying a social mindfulness task. Relative to healthy controls and CHR, spontaneous social mindfulness was reduced in FEP—mirrored by reduced activity in caudate (sensitivity to the rewarding aspects of social mindfulness) and medial PFC (consideration for the other player)—but could be improved when explicitly told to act in another person's best interest.

The comprehensive collection of this T-R-U-S-T Research Topic will not only facilitate, broaden, and improve the current state of the psychoneurobiological signatures of social trust but also bring us a step closer to integrating research findings into a common conceptual framework of reciprocity behavior (including trust and trustworthiness behaviors). Krueger et al. presented an opinion about a neuropsychological framework that explains trust and trustworthiness in the context of reciprocity behaviors—determined by the evaluation of the kindness of a partner's normative action based on the intention as the underlying motivation and the outcome as the consequence of the action, highlining the role of the right anterior insula as a common currency of aversion for determining positive (i.e., norm compliance) and negative (i.e., norm enforcement) reciprocity.

## Author Contributions

FK drafted the editorial. FK and AM-L revised the final submitted version. All authors contributed to the article and approved the submitted version.

## Conflict of Interest

The authors declare that the research was conducted in the absence of any commercial or financial relationships that could be construed as a potential conflict of interest.

